# Metal Release from Microplastics to Soil: Effects on Soil Enzymatic Activities and Spinach Production

**DOI:** 10.3390/ijerph20043106

**Published:** 2023-02-10

**Authors:** Giorgia Santini, Valeria Memoli, Ermenegilda Vitale, Gabriella Di Natale, Marco Trifuoggi, Giulia Maisto, Lucia Santorufo

**Affiliations:** 1 Department of Biology, University of Naples Federico II, Via Cinthia, 80126 Naples, Italy; 2 CeSMA—Centre of Meteorologic and Avanced Thecnology Services, University of Naples Federico II, Nicolangelo Protopisani Course, San Giovanni a Teduccio, 80146 Naples, Italy; 3 Department of Chemistry, University of Naples Federico II, Via Cinthia, 80126 Naples, Italy; 4 BAT Center—Center for Studies on Bioinspired Agro-Environmental Technology, 80100 Naples, Italy

**Keywords:** agroecosystem, microplastics, bio-microplastics, enzymatic activities, soil

## Abstract

Microplastics (MPs) represent emergent pollutants in terrestrial ecosystems. Microplastics can cause the release of metal and damage to crop quality. The present research aimed to evaluate the effects of Mater-bi (Bio-MPs) and polyethylene (PE-MPs) MPs at different concentrations on soil properties and on the growth of *Spinacia oleracea* L. Plants were grown in 30 pots filled with soil mixed with 0.5, 1 and 2% d.w. of Bio-MPs and PE-MPs and in 5 pots filled only with soil, considered as controls (K). At the end of the vegetative cycle, the spinach plants were evaluated for the epigeal (EPI) and hypogeal (HYPO) biomasses and the ratio of HYPO/EPI was calculated. In the soil, the total and the available fractions of Cr, Cu, Ni and Pb and the hydrolase (HA), β-glucosidase (β-glu), dehydrogenase (DHA) and urease (U) activities were evaluated. The results revealed that the addition of Bio-MPs increased soil total Cr, Cu and Pb and available Cu concentrations, and the addition of PE-MPs increased Pb availability. In soil contaminated by both Bio-MPs and PE-MPs, HA and β-glu activities were stimulated, whereas DHA activity was reduced. The HYPO and HYPO/EPI biomasses were reduced only in soils contaminated by the 2% Bio-MPs.

## 1. Introduction

Vegetables are at the base of the human diet as they contain healthy compounds and guarantee human wellbeing [1]. Among the most consumed leafy vegetables worldwide, spinach (*Spinacia oleracea* L.) is easy to grow, has a short growing period and is rich in bioactive compounds that work as reactive-oxygen species scavengers, modulate the expression of genes involved in human metabolism, inflammation, proliferation and provide antioxidant defense [2]. To provide the human population with sufficient vegetables all year round, it is necessary to apply agricultural management promoting and maximizing vegetable production [3].

Plastic mulching is a widespread application in agriculture because it creates soil conditions that favor vegetable growth [4]. For this reason, recently, the use of plastic mulches has increased rapidly worldwide in order to meet the growing demand for food [5]. Unfortunately, plastic mulches are very often left on soil for decades and their improper management causes their degradation [6] in small fragments: microplastics (MPs). Microplastic fragments, especially those that are very tiny, can be absorbed by microorganisms and by plant roots, entering into the food web [7].

Polyethylene (PE), because of its durability, is the most common type of plastic mulch used in agriculture. Recently, in order to mitigate the adverse effects of conventional MPs, biodegradable plastic mulches have been used as they degrade more rapidly than conventional PE film [8], guaranteeing comparable agricultural benefits [9].

Moreover, both conventional and biodegradable MPs can serve as carriers of heavy metals that are added during the production processes to improve the specific performance, functionality and aging properties of the end products [10]. During the weathering and fragmentation processes of plastic sheets, metals can be released leading to the contamination of the soil system [11].

Intensive cultivation and agricultural management have resulted in certain soil problems, among which is soil metal accumulation [12]. Total metal concentration does not reflect metal bioavailability and can exert adverse effects on soil microbial activity [13] and plant growth [14]. In fact, metals present in bioavailable form directly affect plant growth, physiology and development, as plants can easily absorb these elements from soil [15]. Previous research highlighted that in soils contaminated by Cu and Pb an inhibition of seed germination, root proliferation and plant biomass occurred [16].

As with plants, soil microorganisms are also vulnerable to the available fraction of heavy metals that affect their growth and activities [17]. In particular, many soil enzymes (such as hydrolase, dehydrogenase, β-glucosidase and urease) are used as indicators of heavy metal contamination since they quickly respond to changes of soil condition [18]. For example, Hu et al. [19] proposed the use of dehydrogenase as an indicator of microbial activity in soils contaminated by heavy metals.

The effects of heavy metals on soil functioning and plant growth have been recognized [14,20], but the combined effects of metal and MP pollution on microbial activity and plant development are scarcely known. Therefore, the present research aimed to fill the current gap about the impact of MPs and metals on soil activities and on plant growth. Moreover, a comparison of these impacts between soil contaminated by conventional (PE-MPs) and biodegradable microplastics (Bio-MPs) was assessed. To achieve the aims, the research was performed in pots filled with horticultural soils contaminated by PE-MPs and Bio-MPs at three different percentages (0.5%, 1% and 2% *v*/*v*) where individual spinach plants were grown.

## 2. Materials and Methods

### 2.1. Experimental Design

A total of 35 pots with a diameter of 15 cm were set up, each filled with approximately 350 g of horticultural soil (EUROTERRIFLORA s.r.l., Bucine, Italy) (Table 1).

**Figure 1:**
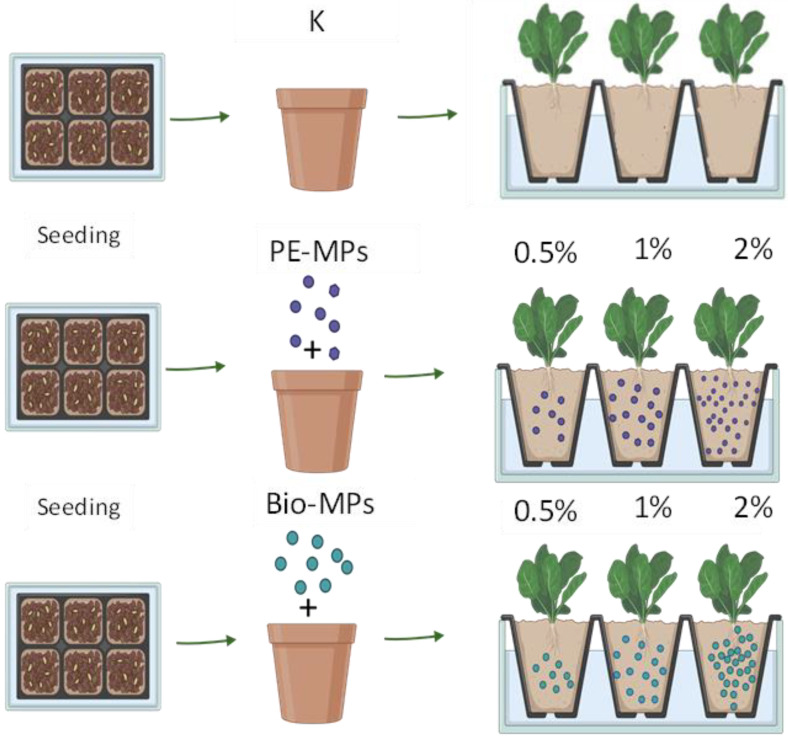
Experimental set-up performed with horticultural soil used as control (K), mixed with conventional microplastics (PE-MPs) and with Mater-bì^®^ microplastics (Bio-MPs) at different percentages (0.5, 1 and 2% of soil dry weight).

Five pots were filled only with horticultural soil and used as controls (K), fifteen were treated with microplastics (MPs) from polyethylene (PE-MPs) at different percentages, 0.5, 1 and 2% of soil dry weight (for 5 pots each), and fifteen were treated with MPs from Mater-bì^®^ (Bio-MPs) at the same percentages of PE-MPs (Figure 1). The chosen percentages are reported in the literature as the most used to highlight the impacts of MPs on soil properties and plants [7,21].

**Figure 2:**
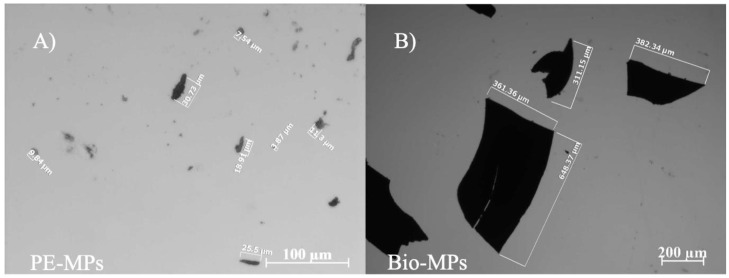
Images showing (**A**) conventional (PE) and (**B**) Mater-bì^®^ (Bio) microplastic (MP) fragments, obtained to perform the experiment.

Fragments of PE and Mater-bì^®^ were generated in the laboratory from agricultural mulch sheets using a liquid nitrogen grinder. Grinding cycles were performed from 5 to 10 times to achieve microplastic sizes ranging from 20 μm to 5 mm in diameter (Figure 2A,B).

Previously, forty seeds of *Spinacia oleracea* L. (spinach) of the “matador” variety were germinated in the dark at a room temperature of 24 ± 1 °C. Then, the seedlings were transplanted in pots as described above and grown in a greenhouse at the same environmental conditions: PPFD of 900 ± 100 µmol (photons) m^−2^ s^−1^ at the top of the canopy, photoperiod of 12 h, temperature of 26 ± 1 °C, relative humidity of 55–60%. Plants were regularly watered and followed until the end of the vegetative cycle. Spinach was chosen for its importance at social and economic levels. In fact, it is widely cultivated in southern Italy as it is at the base of the human Mediterranean diet.

### 2.2. Sampling and Analyses

The soil and plant sampling were carried out at the end of plant vegetative cycle. In each pot, the spinach plants were collected removing the soil from the roots and separating the epigeal (EPI) from the hypogeal (HYPO) portions. Contextually, soil samples (0–10 cm) were collected from each pot.

The total concentrations and available fractions of Cr, Cu, Ni and Pb were measured according to Memoli et al. (2017) [22] and measured by inductively coupled plasma mass spectrometry (ICP-MS Aurora M90, Bruker, Billerica, MA, USA).

Hydrolase activity (HA) was determined by adding 7.5 mL of 60 mM potassium phosphate (pH 7.6) and 0.100 mL of fluorescein diacetate (FDA) to 3 g of fresh soil. The details of the method were reported in Adam and Duncan (2001) [23].

Dehydrogenase activity (DHA) was determined by adding 1 mL of 1.5% 2,3,5-triphenyltetrazolium chloride (TTC) dissolved in 0.1 M Tris-HCl buffer (pH 7.5) to 1 g of fresh soil according to Memoli et al. (2018) [17].

β-glucosidase activity (β-glu) was determined by adding 4 mL of modified universal buffer (MUB) pH 6 and 1 mL of 0.025M p-nitrophenyl β-D-glucopiranoside (PNP) to 1 g of soil according to Tabatabai and Bremner (1969) and Tabatabai (1988) [24,25].

Urease activity (U) was determined by adding 0.5 mL of urea (0.1 M) and 4 mL of borate buffer (0.1 M pH 8.8) to 1 g of fresh soil according to Kendeler (1988) and Alef and Nannipieri (1995) [26,27].

The biomass of EPI and HYPO portions was determined on oven-dried plant samples at 75 °C for 48 h and expressed in grams of dry weight (d.w.) per plant. The ratio of HYPO/EPI for all experimental conditions was also calculated.

### 2.3. Statistical Analyses

In order to verify the normal data distribution and homogeneity of variance, the Shapiro–Wilks and Levene Median tests were assessed, respectively.

The differences in soil element concentrations (total and available), in soil enzymatic activities (HA, DHA, β-glu and U) and in plant biomasses (EPI, HYPO, HYPO/EPI), between K and the different percentages (0.5, 1 and 2% d.w.) of PE-MPs and Bio-MPs were assessed through one-way analysis of variance (ANOVA) combined with post hoc comparison tests (pairwise Student–Newman–Keuls test or Fisher LSD method).

The differences in soil element concentrations (total and available), in soil enzymatic activities (HA, DHA, β-glu and U) and in plant biomasses (EPI, HYPO, HYPO/EPI), among the percentages (0.5, 1 and 2% d.w.) inside the same treatment (PE-MPs or Bio-MPs) were assessed through one-way analysis of variance (ANOVA) combined with post hoc comparison tests (pairwise Student–Newman–Keuls test or Fisher LSD method).

The differences in soil element concentrations (total and available), in soil enzymatic activities (HA, DHA, β-glu and U) and in plant biomasses (EPI, HYPO, HYPO/EPI), between the same percentage (0.5, 1 and 2% d.w.) of PE-MPs and Bio-MPs were assessed through the *t*-test.

A Principal Components Analysis (PCA) was performed on soil and plant properties to evaluate the treatment distribution (K, PE-MPs and Bio-MPs) and to identify the main properties driving the distribution. The PCA was conducted using the Past 4.0 software. The PERMANOVA analyses (Vegan package, Adonis function—pairwise.perm.manova test for *p* < 0.05) were carried out on the selected soil and plant properties to highlight the significant differences among treatments (K, PE-MPs and Bio-MPs).

The statistical analyses and the PERMANOVA analyses were performed using the R 4.0.3 programming environment and graphical displays with Sigma-Plot 9.0 software (Jandel Scientific, San Rafael, CA, USA).

## 3. Results

### 3.1. Soil Metal Total Concentrations

**Figure 3:**
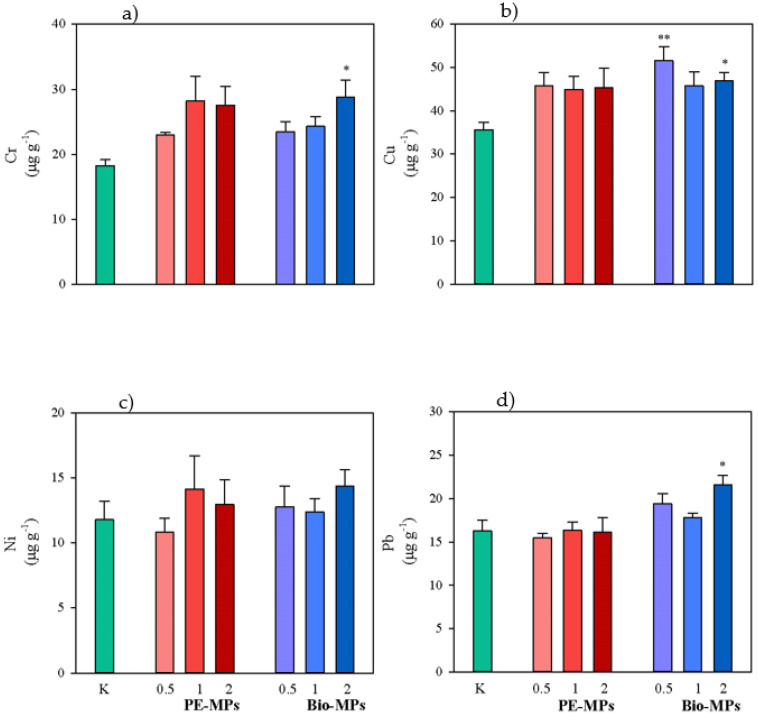
Mean values (±s.e.) of total concentrations of Cr (**a**), Cu (**b**), Ni (**c**) and Pb (**d**) measured in soils without microplastics (K), mixed with Polyethylene (PE-MPs) and Mater-bì^®^ (Bio-MPs) microplastics at different percentages (0.5, 1 and 2% d.w.). Asterisks indicate significant differences between soils mixed with microplastics and control (one-way ANOVA; *p* < 0.05).

Total concentrations of Cr, Cu, Ni and Pb in control (K) and in soils contaminated with different percentages (0.5, 1 and 2% d.w.) of conventional microplastics (PE-MPs) and biodegradable microplastics (Bio-MPs) are reported in Figure 3. Soil total Ni concentrations in both PE-MPs and Bio-MPs did not statistically vary as compared to K (Figure 3). Instead, soil total concentrations of Cr, Cu and Pb in 2% Bio-MPs (Cr: 28.7 μg g^−1^ d.w.; Cu: 46.9 μg g^−1^ d.w.; Pb: 21.6 μg g^−1^ d.w.) were significantly (*p* < 0.05) higher than in K (Cr: 19.9 μg g^−1^ d.w.; Cu: 35.6 μg g^−1^ d.w.; Pb: 16.2 μg g^−1^ d.w.) and also soil total concentrations of Cu in 0.5 % Bio-MPs (51.5 μg g^−1^ d.w.) were significantly (*p* < 0.01) higher than in K (Figure 3).

Total concentrations of the investigated metals did not statistically vary among the different percentages within the same treatment (PE-MPs or Bio-MPs).

Yet, the total concentrations of the investigated metals in soils contaminated with the same percentage did not statistically vary between the two treatments (PE-MPs vs. Bio-MPs), except for Pb, which was significantly (*p* < 0.05) higher in 2% PE-MPs than 2% Bio-MPs (Table 2).

### 3.2. Soil Available Fractions

**Figure 4:**
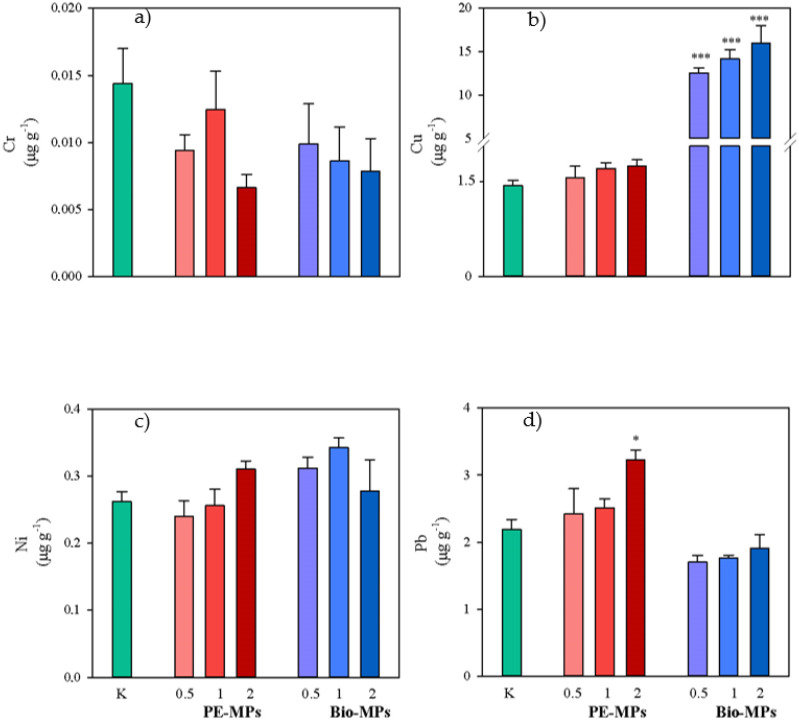
Mean values (±s.e.) of availability of Cr (**a**), Cu (**b**), Ni (**c**) and Pb (**d**) measured in soils without microplastics (K), mixed with Polyethylene (PE-MPs) and Mater-bì^®^ (Bio-MPs) microplastics at different percentages (0.5, 1 and 2% d.w.). Asterisks indicate significant differences between soils mixed with microplastics and control (one-way ANOVA; *p* < 0.05).

The available fractions of Cr, Cu, Ni and Pb in control (K) and in soils contaminated with different percentages (0.5, 1 and 2% d.w.) of conventional microplastics (PE-MPs) and biodegradable microplastics (Bio-MPs) are reported in Figure 4. Soil Cr and Ni availabilities in both PE-MPs and Bio-MPs did not significantly vary as compared to K (Figure 4). Instead, soil Cu availabilities were significantly (*p* < 0.001) higher in all Bio-MP treatments (0.5%: 12.5 μg g^−1^ d.w.; 1%: 14.2 μg g^−1^ d.w.; 2%: 15.9 μg g^−1^ d.w.) than in K (1.39 μg g^−1^ d.w.) (Figure 4) and also soil Pb availabilities were significantly (*p* < 0.05) higher in 2% PE-MPs (3.23 μg g^−1^ d.w.) than in K (2.18 μg g^−1^ d.w.) (Figure 4).

Metal availabilities did not significantly vary among the different percentages within the same treatment (PE-MPs or Bio-MPs).

The comparison of metal availabilities in soils contaminated with the same percentage of PE-MPs and Bio-MPs highlighted that Cr did not significantly vary (Table 2). Instead, Cu availabilities in all the percentage Bio-MPs were significantly (*p* < 0.001 for 0.5% and 1% and *p* < 0.01 for 2%) higher than in PE-MPs (Table 2); Ni availabilities in 0.5% and 1% Bio-MPs were significantly (*p* < 0.001 and *p* < 0.05, respectively) higher than in PE-MPs (Table 2). By contrast, Pb availabilities in 1% and 2% PE-MPs were significantly (*p* < 0.05) higher than in Bio-MPs (Table 2).

### 3.3. Ratio of Metal Availability with Respect to Total Concentration

The ratios between the availability and the total concentration for Cr, Cu, Ni and Pb are reported in Table 3. The ratios of all the metals for PE-MPs as well as those of Cr and Ni for Bio-MPs did not significantly vary as compared to K (Table 3). Instead, the ratios of Cu for all the percentage Bio-MPs were significantly (*p* < 0.05) higher than in K (Table 3), those of Pb for 0.5% and 2% Bio-MPs were significantly (*p* < 0.05) lower than in K and those of Pb for 2% PE-MPs were significantly (*p* < 0.05) higher than in K (Table 3).

The ratios between the availability and the total concentration for the investigated metals did not significantly vary among the different percentages within the same treatment (PE-MPs or Bio-MPs).

The ratios in soils contaminated with the same percentage did not significantly vary between the two treatments (PE-MPs vs. Bio-MPs) for Cr and Ni (Table 4). Instead, the ratios for Cu were significantly higher in Bio-MPs than in PE-MPs for all the percentages (*p* < 0.01 for 0.5% and *p* < 0.001 for 1% and 2%), whereas the ratios for Pb were significantly higher in PE-MPs than in Bio-MPs for 1% and 2% (*p* < 0.01) (Table 4).

### 3.4. Enzymatic Activities in Soil Contaminated with PE-MPs and Bio-MPs at Different Percentages

**Figure 5:**
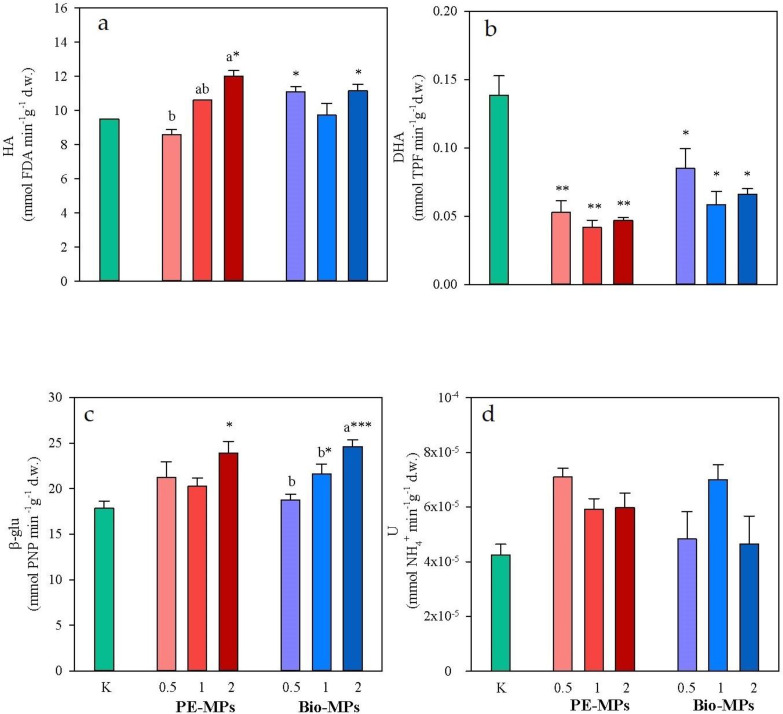
Mean values (±s.e.) of hydrolase (HA, (**a**)), dehydrogenase (DHA, (**b**)), β-glucosidase (β-glu, (**c**)) and Urease (U, (**d**)) activities measured in soil without microplastics (K), mixed with Polyethylene (PE) and Mater-bì^®^ (Bio) microplastics at different concentrations (0.5, 1 and 2% d.w.). Asterisks indicate significant differences between soils mixed with microplastics and control, respectively (one-way ANOVA; *p* < 0.05). Different small letters indicate significant differences among the percentages of the same treatment (one-way ANOVA; *p* < 0.05).

The soil enzymatic activities, hydrolase (HA), dehydrogenase (DHA), β-glucosidase (β-glu) and urease (U), are reported in Figure 5.

Urease in both PE-MPs and Bio-MPs did not significantly vary as compared to K (Figure 5). Instead, HA and β-glu in 2% PE-MPs (HA: 10.5 mmol FDA min^−1^g^−1^ d.w. and β-glu: 23.8 mmol PNP min^−1^g^−1^ d.w.) were significantly (*p* < 0.05) higher than in K (HA: 8.84 mmol FDA min^−1^g^−1^ d.w. and β-glu: 17.8 mmol PNP min^−1^g^−1^ d.w) (Figure 5); by contrast, DHA in all the percentage PE-MPs (0.5%: 0.06 mmol TPF min^−1^g^−1^ d.w.; 1%: 0.06 mmol TPF min^−1^g^−1^ d.w.; 2%: 0.05 mmol TPF min^−1^g^−1^ d.w.) was significantly (*p* < 0.01) lower than in K (0.139 mmol TPF min^−1^g^−1^ d.w.) (Figure 5). Moreover, HA in 0.5% and 2% Bio-MPs (0.5%: 10.9; 2%: 10.4) was significantly (*p* < 0.05) higher than in K (Figure 5); β-glu in 1% and 2% Bio-MPs (1%: 21.6 mmol PNP min^−1^g^−1^ d.w.; 2%: 25.6 mmol PNP min^−1^g^−1^ d.w.) was significantly (*p* < 0.05 and *p* < 0.001, respectively) higher than in K (Figure 5); DHA in all the percentage Bio-MPs (0.5%: 0.10 mmol TPF min^−1^g^−1^ d.w.; 1%: 0.07 mmol TPF min^−1^g^−1^ d.w.; 2%: 0.10 mmol TPF min^−1^g^−1^ d.w.) was significantly (*p* < 0.05) lower than in K.

The enzymatic activities of DHA and U in soils did not significantly vary among the different percentages within the same treatment (PE-MPs or Bio-MPs) except for HA for PE-MPs and β-glu for Bio-MPs that were higher at the increase in the percentage of MPs (Figure 5).

The enzymatic activities in soils contaminated with the same percentage did not significantly vary between the two treatments (PE-MPs vs. Bio-MPs).

### 3.5. Biomasses of Plants

**Figure 6:**
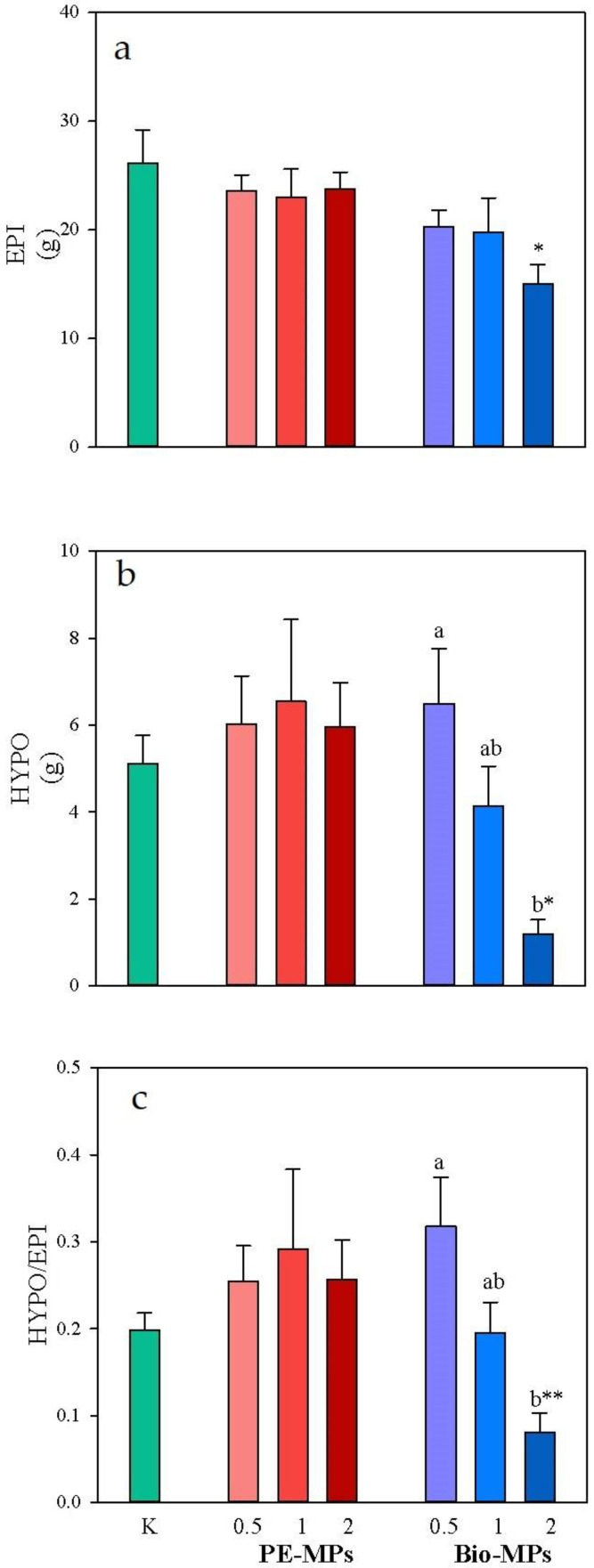
Mean values (±s.e.) of (**a**) epigeal (EPI), (**b**) hypogeal (HYPO) and (**c**) hypogeal and epigeal ratio (HYPO/EPI) of spinach plants grown in soil without microplastics (K), mixed with Polyethylene (PE) and Mater-bì^®^ (Bio) microplastics at different concentrations (0.5, 1 and 2% d.w.). Asterisks indicate significant differences between soils mixed with microplastics and control, respectively (one-way ANOVA; *p* < 0.05). Different small letters indicate significant differences among the percentages of the same treatment (one-way ANOVA; *p* < 0.05).

The values of plant epigeal (EPI) and hypogeal (HYPO) biomasses and their ratios (HYPO/EPI) measured at the end of the vegetative cycle of plants grown on control soils and in soils contaminated with different percentages of conventional microplastics and biodegradable microplastics are reported in Figure 6. 

The comparison of the biomasses of plants grown on K, PE-MP and Bio-MP soils highlighted that both EPI and HYPO biomasses as well as the HYPO/EPI of plants grown on soils contaminated by PE-MPs did not significantly vary as compared to those of plants grown on K (Figure 6); instead, they were significantly (*p* < 0.05) lower in plants grown on 2% Bio-MPs (EPI: 15.0 g; HYPO: 1.2 g; HYPO/EPI: 0.1 g) than in K (EPI: 26.1 g; HYPO: 5.1 g; HYPO/EPI: 0.2 g) (Figure 6).

The comparison of the biomasses of plants grown on soils at different percentages of PE-MPs highlighted that no statistical differences were found. Instead, EPI, HYPO and HYPO/EPI were significantly lower at the increase in the percentage of Bio-MPs (Figure 6).

The comparison of EPI and HYPO biomasses and the HYPO/EPI ratio of plants grown at the same percentage between the two treatments (PE-MPs vs. Bio-MPs) highlighted that no significant differences were found (Figure 6).

### 3.6. Correlations between Soil Abiotic and Biotic Parameters in Soil Contaminated with PE-MPs and Bio-MPs

**Figure 7:**
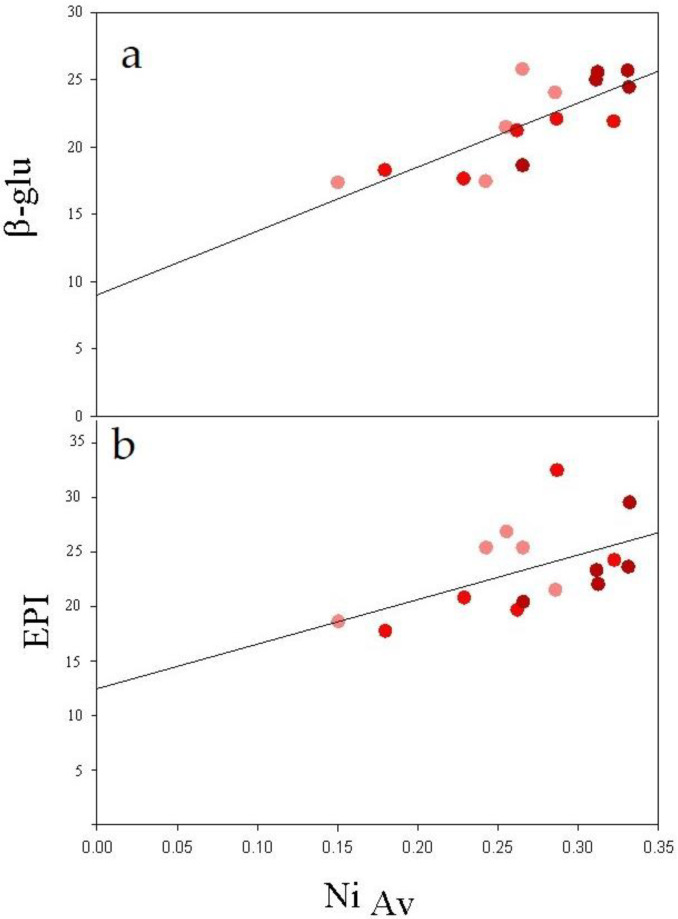
Regression lines (Spearman’s correlations) between β-glucosidase activity (β-glu, (**a**)) and plant epigeal biomass (EPI, (**b**)) of spinach plants with Ni available fractions in soil contaminated by PE-MPs (0.5%: light red bar; 1%: red bar and 2%: dark red bar).

The correlations performed to evaluate the significance of the relationships between the enzymatic activities in soils or the plant biomasses and the soil metal concentrations highlighted that, for soils contaminated with PE-MPs, both β-glu and plant epigeal biomass (EPI) were positively correlated to Ni availability (Figure 7).

### 3.7. Principal Component Analyses on Soil Parameters

**Figure 8:**
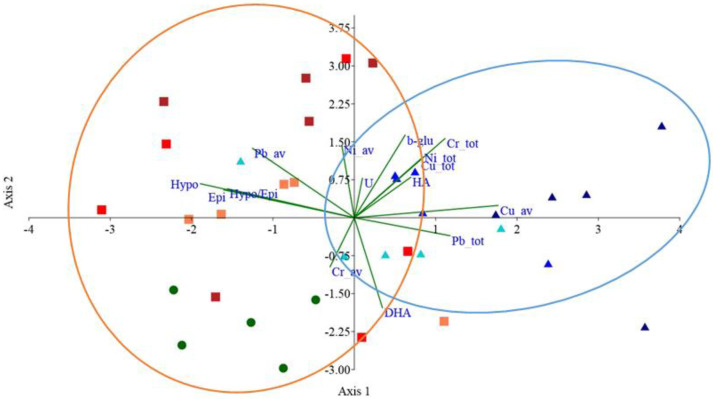
Graphical display of the first two axes of the Principal Component Analysis (PCA) on the soil total and available Cu, Cr, Ni and Pb concentrations, enzymatic activities (HA, DHA, β-glu and U) and plant biomasses in soil without microplastics (green dots), mixed with 0.5, 1 and 2% d.w. of Polyethylene (orange, red and dark red squares, respectively) and of Mater-bì^®^ (cyan, blue and dark blue triangles, respectively) microplastics. Significant differences among the treatments were shown by confidence ellipses (PERMANOVA analysis *p* < 0.05).

The PCA, performed on all the investigated soil and plant properties, highlighted that the first two axes accounted, respectively, for 20% and 17% of the total variance (Figure 8). The available fraction of Cu, EPI, HYPO and the HYPO/EPI ratio explained the major part of the variance of the first axis (Figure 8), whereas the available fractions of Ni and Pb and the total metal concentration of Cr, DHA and β-glu explained the major part of the variance of the second axis (Figure 8). The first axis clearly separated PE-MPs and Bio-MPs, as the former located along the negative part of the axis and the latter along the positive one (Figure 8). The second axis clearly separated K soils as they located along the negative part of the second axis (Figure 8). The PERMANOVA analyses highlighted that soil treated with Bio-MPs was significantly different (*p* < 0.05) from K and PE-MP soils.

## 4. Discussion

The present research highlighted that the addition of conventional microplastics (PE-MPs) and biodegradable microplastics (Bio-MPs) to soils caused variations in the total and available fraction of the investigated metals (Cu, Cr, Ni and Pb) that often were also significant. The findings agree with those reported by several researchers [28,29,30] who found that MPs can affect the speciation, transformation and bioavailability of heavy metals such as Zn, Cu, Ni, Cd, Cr, As and Pb.

Contrarily from what happened in soils contaminated by PE-MPs, those highly (2%) contaminated by Bio-MPs caused significant increases in total concentrations of Cu, Cr and Pb. This could be due to the release in soils of harmful additives, containing metals used during the production of bioplastic films [31,32,33]. Bioplastics have stronger metal adsorption capacities than conventional plastics, due to their crystallization and carrier adsorption characteristics [11]. In addition, this phenomenon becomes more marked at the highest concentrations of biodegradable microplastics, because of the high contents of fragments that increase the contact surface area between soil and biodegradable microplastics. Finally, the phenomenon of metal adsorption to soil particles cannot be neglected.

Although the soils contaminated by Bio-MPs showed higher total concentrations of Cr, Cu and Pb, as compared to K, only the Cu availability significantly increased. In fact, Cu can lead to chemical speciation through physical, chemical and biological interactions with soil components [34]. By contrast, although the soils contaminated by PE-MPs did not show significant differences in total concentrations of the investigated metals, as compared to K, an increase in Pb availability was observed. It can be supposed there is a release of petroleum-based compounds containing Pb by conventional plastic films [30]. The different behaviors of Cu and Pb were confirmed by the calculated ratios between the availability and the total concentration of these metals that were, respectively, higher in soils contaminated by Bio-MPs and PE-MPs.

Among the investigated soil enzymatic activities, only U did not appear to be affected by MPs, whereas HA and β-glu were stimulated and DHA reduced by the presence of both PE-MPs and Bio-MPs (although not at all the tested percentages). The effects of microplastics on microbial activity are highly variable and dependent on the kind and concentration of microplastic [35]. The observed stimulation of HA and β-glu agrees with several pieces of research [36,37] and could be due to the possible release of dissolved carbon from plastic films in soil [38]. This hypothesis is corroborated by the increase in β-glu, using carbon compounds as a substrate [39], already at 1% Bio-MPs. Instead, the reduction of the intracellular enzyme DHA in both PE-MP- and Bio-MP-contaminated soils suggests an overall stress condition for microbial metabolism [40,41].

The effect of MPs on the investigated crop was limited to highest percentages of Bio-MPs that inhibited plant growth, as significant reductions of both HYPO and EPI biomasses were observed. The findings agree with those reported by various researchers who found negative dose-effect impacts on plant growth [7,42,43] due to MPs. Also, Qi et al. [44] found that starch-based Bio-MPs had a negative effect on wheat biomass compared to PE-MPs. The decrease in HYPO biomass and the HYPO/EPI ratio at the increase in percentage of Bio-MPs suggests that these plastics hinder the movement of water and nutrients in soil, limiting their absorption and utilization, with a negative consequence on plant root growth [45,46].

In PE-MP-contaminated soils a key role was played by Ni availability, which enhanced EPI biomass and β-glu activity. The lowest Ni availability, compared to those measured in Bio-MP-contaminated soils, suggests that this metal is present in concentrations essential for crop growth and for maintaining its health [47].

An overall evaluation, considering the investigated soil properties and the crop biomasses, highlighted a clear separation of Bio-MP-contaminated soils from both PE-MPs and K. Bio-MP-contaminated soils, especially at 2%, were characterized by high Cu availability and reduced crop production, indicating its role in metal contamination increase and the inhibition of plant biomasses.

## 5. Conclusions

The findings contributed to highlight differences in soil properties and crop production after soil MP contamination. In particular, the addition of PE-MPs did not cause variations in soil total metal concentrations as compared to K, whereas the addition of Bio-MPs caused the increase in total Cr, Cu and Pb concentrations. Notwithstanding, in soils contaminated by PE-MPs higher Pb availability was observed, and in soils contaminated by Bio-MPs only the Cu availability significantly increased as compared to K.

Extracellular enzymatic (HA and β-glu) activities were stimulated in MP-contaminated soils, especially at 2%, whereas the intracellular one (DHA) was reduced. Finally, the HYPO and HYPO/EPI biomasses were reduced only in soils contaminated by the highest percentage Bio-MPs.

Based on the obtained data, it can be concluded that Bio-MPs more than PE-MPs contribute to a major metal release in soils and have a negative impact on spinach biomass.

The present research highlighted a negative role of Bio-MP presence in soils, but further studies in open fields are required to clarify the effects of Bio-MPs on soil properties and plant growth.

## Figures and Tables

**Table 1:** Properties of horticultural soil (EUROTERRIFLORA s.r.l.) used to perform the experiment with MPs and spinach (*Spinacia oleracea* L.).

Horticultural Soil Properties	
pH	7
Electrical conductivity	0.8 dS/m
Carbon	30%
Dry bulk density	360 kg/m^3^
Total porosity	80% (*v*/*v*)

**Table 2:** Significant differences in total and available Cu, Cr, Ni and Pb concentrations within the pots with the same Polyethylene (PE) and Mater-bì^®^ (Bio) microplastic concentrations (*t*-test; *** *p* < 0.001; ** *p* < 0.01; * *p* < 0.05).

	PE-MPs vs. Bio-MPs		
	0.5%	1%	2%
Cr_Tot_	n.s.	n.s.	n.s.
Cu_Tot_	n.s.	n.s.	n.s.
Ni_Tot_	n.s.	n.s.	n.s.
Pb_Tot_	n.s.	n.s.	*
Cr_Av_	n.s.	n.s.	n.s.
Cu_Av_	***	***	**
Ni_Av_	***	*	n.s.
Pb_Av_	n.s.	**	**

**Table 3:** Mean values (±s.e.) of available fraction and total concentration ratios of Cu, Cr, Ni and Pb calculated in soil without microplastics (K), mixed with Polyethylene (PE) and Mater-bì^®^ (Bio) microplastics at different concentrations (0.5, 1 and 2% d.w.). Asterisks indicate significant differences between soils mixed with microplastics and control, respectively (one-way ANOVA; *p* < 0.05).

	K	PE-MPs			Bio-MPs		
		0.5%	1%	2%	0.5%	1%	2%
Cr	0.10	0.04	0.07	0.03	0.04	0.04	0.03
Cu	3.93	3.26	3.76	3.92	24.7 *	31.4 *	34.2 *
Ni	2.38	2.26	2.10	2.60	2.73	2.83	2.05
Pb	13.7	14.5	15.8	20.9 *	8.90 *	9.95	8.99 *

**Table 4:** Significant differences in available fraction and total concentration ratios of Cu, Cr, Ni and Pb within the pots with the same Polyethylene (PE) and Mater-bì^®^ (Bio) microplastic concentrations (*t*-test; *** *p* < 0.001; ** *p* < 0.01).

	PE-MPs vs. Bio-MPs		
	0.5%	1%	2%
Cr	n.s.	n.s.	n.s.
Cu	**	***	***
Ni	n.s.	n.s.	n.s.
Pb	n.s.	**	**

## Data Availability

The data that have been used are confidential.
